# The transbasal approach to the anterior skull base: surgical outcome of a single-centre case series

**DOI:** 10.1038/s41598-020-80255-8

**Published:** 2020-12-31

**Authors:** A. Kaywan Aftahy, Melanie Barz, Arthur Wagner, Julia S. Bermeitinger, Claire Delbridge, Chiara Negwer, Bernhard Meyer, Jens Gempt

**Affiliations:** 1 Department of Neurosurgery, Medical Faculty, School of Medicine, Klinikum Rechts Der Isar, Technical University Munich, Ismaninger Str. 22, 81675 Munich, Germany; 2 Department of Neuropathology, Institute of Pathology, School of Medicine, Klinikum Rechts Der Isar, Technical University Munich, Munich, Germany

**Keywords:** Outcomes research, Brain, Head and neck cancer, Surgical oncology

## Abstract

A variety of sinonasal tumours, meningiomas or other lesions of the anterior skull base involve the paranasal sinuses and the periorbital area. The transbasal approach (TBA) has turned out to be a feasible technique to reach those lesions. A retrospective review at a neurosurgical university department between November 2007 and January 2020 with adult patients who underwent resection of oncologic pathologies through TBA. Surgical technique, extent of resection (EOR), clinical outcome and postoperative complications were analysed. 18 TBAs between November 2007 and January 2020 were performed. Median age was 62 (range 25–83), 7 female and 11 male patients. Gross total resection rate was 85.8% throughout all entities. Four (22.2%) patients suffered from WHO°I meningiomas and 14 (77.7%) from other extra-axial lesions. Preoperative Karnofsky Performance Status Scale (KPSS) was 80% (range 40–90), postoperative KPSS 80% (range 20–100). Rate of postoperative complications requiring intervention was 16.7%. Median follow-up was 9.8 (range 1.2–71.8) months. Modifications and extensions of the classic TBA are not mandatory. Complete resection can be performed under functional and cosmetic-preserving aspects. Second-step procedures such as transnasal approaches may be performed to avoid high morbidity of more aggressive TBAs, if necessary. Surgical considerations should be kept simple and straightforward.

## Introduction

Oncologic lesions in the anterior skull base with transbasal infiltration and involvement of the paranasal sinuses and orbit have always been surgically challenging due to the anatomic relationships in this region. These skull base tumours can originate from a variety of structures such as paranasal sinuses, nasal pharynx, leptomeninges and orbital or cranial nerves, as well as from the squamous cells or the glands^1,2^.

The transbasal approach (TBA) turned out to be an advanced but feasible approach to expose deep-lying lesions of the anterior skull base.

Since its first usage and systematic description by Frazier, Tessier, Derome and Dandy^3–6^, several variations, extensions and alternatives have been described and discussed in the last decades by technical notes, cadaver studies and small sample series^1,7–12^. Modifications have been made to the original technique such as removal of the supraorbital bar, ethmoidectomy, sphenoidectomy and extradural resection of the clivus.

Beals and Joganic tried to incorporate TBAs but could not applicate sufficiently to the current literature^13^. Therefore, Feiz-Erfan et al. categorized and proposed a classification for the TBA to facilitate the comparison and discussion of these approaches^5^.

However, due to greater technical variety, rarity and technically demanding nature of the TBA, only few larger series such as Liu’s, Lang’s, Raso’s or Barzaghi’s series with 15, 18, 20 and 21 patients, respectively, exist regarding surgical considerations, perioperative morbidity/mortality, follow-up and outcome^1,14–18^.

This study aims to highlight the sufficiency of simple TBAs without further modifications and osteotomies and thus the reduction of perioperative morbidity. Researchers have accepted little pathological heterogeneity to discuss this problem from a surgical and technical point of view.

## Materials and methods

### Study population and clinical parameters

We performed a retrospective review of our clinical database. From November 2007 until January 2020, we screened the clinical documentation files and neuropathological records of adult patients (≥ 18 years old) who underwent surgery through a TBA for extra-axial oncologic lesions. Eighteen patients could be identified who were operated with this technique.

We analysed clinical patient files for neurological symptoms, Karnofsky Performance Status Scale (KPSS), postoperative new permanent neurological deficits, postoperative complications and re-interventions. We defined the total rate of postoperative new deficits/complications/reinterventions as the number of events divided by the number of patients. Then, we recorded the type of TBA (according to Feiz-Erfan et al.)^5^ and histopathological entity. Radiological outcome parameters consisted of anatomic location as well as the extent of resection (EOR) according to postoperative cranial magnetic resonance imaging (MRI).

### Statistical evaluation

We performed descriptive statistical analysis using STATA Version 13.1 (2011, StataCorp, College Station, Texas).

In descriptive data analysis, non-normally distributed data are shown as median and interquartile range (IR), and normally distributed variables are shown as mean and standard deviation.

### Surgical technique

Previous publications^5,6,11,19^ have described in detail the operative techniques and nuances for the classic TBA. In fact, we did not use any noteworthy alterations of known techniques, but we advocate regular usage of a navigation system, if applicable, for individually optimized craniotomy. As already mentioned, we just performed level 0 TBAs according to Feiz-Erfan et al.^5^.

The patient is placed in the supine position with the head slightly extended or flexed based on the vertical tumor extent. The head position mobilizes the frontal lobes away using gravity. The head typically does not require rotation for the transbasal lesions. A bicoronal incision is performed. This incision should be as anterior as possible while staying behind the hairline. The incision may not always has to to reach the tragus, but at least the level of the superior edge of the pinna. The extent of the head flexion is tailored based on the cranial extension and inferior reach of the lesion into the nasal sinuses. The scalp incision should leave the pericranium, temporalis muscle, and fascia intact. We elevate a vascularized galea-periosteum graft as a separate layer. This vascularized graft will be reflected and based anteriorly along the orbital rims. The flap is elevated, stretched, and kept moist during the operation by covering it with a wet piece of sponge. A bifrontal craniotomy along the orbital roofs follows. Burr holes in the forehead should be avoided for cosmetic reasons. The vertical height of the craniotomy is under 10 cm in most cases, however, we prefer large bone flaps for satisfying exposure and, if necessary, better cerebrospinal fluid release and further frontal lobe relaxation. Extradural dissection and lobar elevation away from the Crista Galli and exposure of the cribiform plate are performed. The dura is mobilized from the orbital roof and Crista Galli. Sharp delicate dissection is used over the olfactory bulb to disconnect the bulb and expose possible anterior extradural extension of the transbasal lesion.

Upon elevation of the bone flap, the frontal sinus mucosae are thoroughly removed and filled with antiseptic sponges Transbasal lesions need full exposure. In such cases, besides cranialization of the sinus, meticulous dural reconstruction and skull base coverage must be performed, whereas we prioritize usage of a galea-periosteum flap. We moved away from using fascia lata in the first place as we experienced more CSF leaks than with the flap, which may be based on insufficient vascularization. Necrosis and thus also source of superinfections may be further consequences. However, this just reflects our own institutional experience, whereas techniques of anterior skull base reconstruction have been extensive discussed and algorithms proposed^20–22^.

### Ethics approval

The local ethics committee of the Technical University Munich, School of Medicine (231/20 S-EB) (Ethikkommission der Fakultät für Medizin der Technischen Universität München), approved our study. We conducted it in accordance with the ethical standards of the 1964 Declaration of Helsinki and its later amendments^23^.

The requirement for written informed consent was waived by the ethics committee (Ethikkommission der Fakultät für Medizin der Technischen Universität München).

## Results

### Patient population

**Figure 1:**
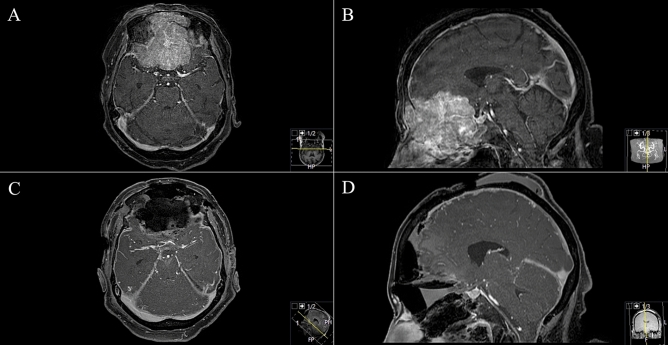
Exemplary case 1: a 74-year-old female patient admitted after fall at home and back pain. Cranial routine CT scan displayed a huge transbasal bone infiltrating and destroying a tumor with sinonasal affection. (**A**,**B**) Preoperative T1-weighted gadolinium-enhanced MR imaging showing sinonasal and orbit-infiltrating tumor with subtotal anterior skull base destruction. (**C**,**D**) Postoperative MR control showing successful resection. Reconstruction was performed with split-bone graft and galea-periosteum flap. Pathologic findings revealed adenoid cystic carcinoma. The patient recovered well from the procedure; she underwent VP-shunt implantation in further course.

Eighteen patients underwent surgical resection through a TBA between November 2007 and January 2020. Median patient age at time of surgery was 62 (range: 25–83) years. Seven (38.9%) female and eleven (61.1%) male patients were included: five (27.8%) were asymptomatic, and tumours were discovered incidentally during routine check-ups, diagnostic workup for sinusitis or, as in exemplary case 1, after a fall at home (Fig. 1); and 13 (72.2%) patients were symptomatic, which included cephalgia, headache, cranial nerve deficits (amaurosis/anosmia) and other symptoms. Median preoperative KPS was 80% (40–90), and median postoperative KPS was 80% (range 20–100). Table 1 provides further detailed demographic and clinical information. Preoperative anosmia was prevalent in 44.4% (8) of the patients, and preoperative visual deficits was prevalent in 22.2% (4).

**Table 1:** Demographics, preoperative and tumor characteristics.

Demographics, % (N) or mean/median (SD/range)	Total (18)
Age	62 (25–83)
Sex	M 61.1% (11)
	F 38.9 (7)
**Clinical presentation % (N)**	
Preoperative KPS	80 (40–90)
Asymptomatic	27.8% (5)
Visual deficits/ Anopsia	5.6% (1)
Amaurosis	22.2% (4)
Headache	44.4% (8)
Concentration/ memory/ cognition impairment	5.6% (1)
Psychomotoric disorders/ dizziness/ vigilance	5.6% (1)
Breathing problems	16.6% (3)
Vertigo	5.6% (1)
Exophthalmos	11.2% (2)
Diplopia	5.6% (1)
Epistaxis	5.6% (1)
Rhinorrhoea/epiphora	5.6% (1)
**Tumor entity % (N)**	
Meningioma	22.2% (4)
Metastasis	5.6% (1)
Adenoid cystic carcinoma	16.7% (3)
Invasive squamous cell carcinoma	5.6% (1)
Papillary transitional carcinoma	5.6% (1)
Sinonasal intestinal adenocarcinoma	16.7% (3)
Esthesioneuroblastoma	27.8% (5)

### Tumor entities, location and approach-related findings

**Figure 2:**
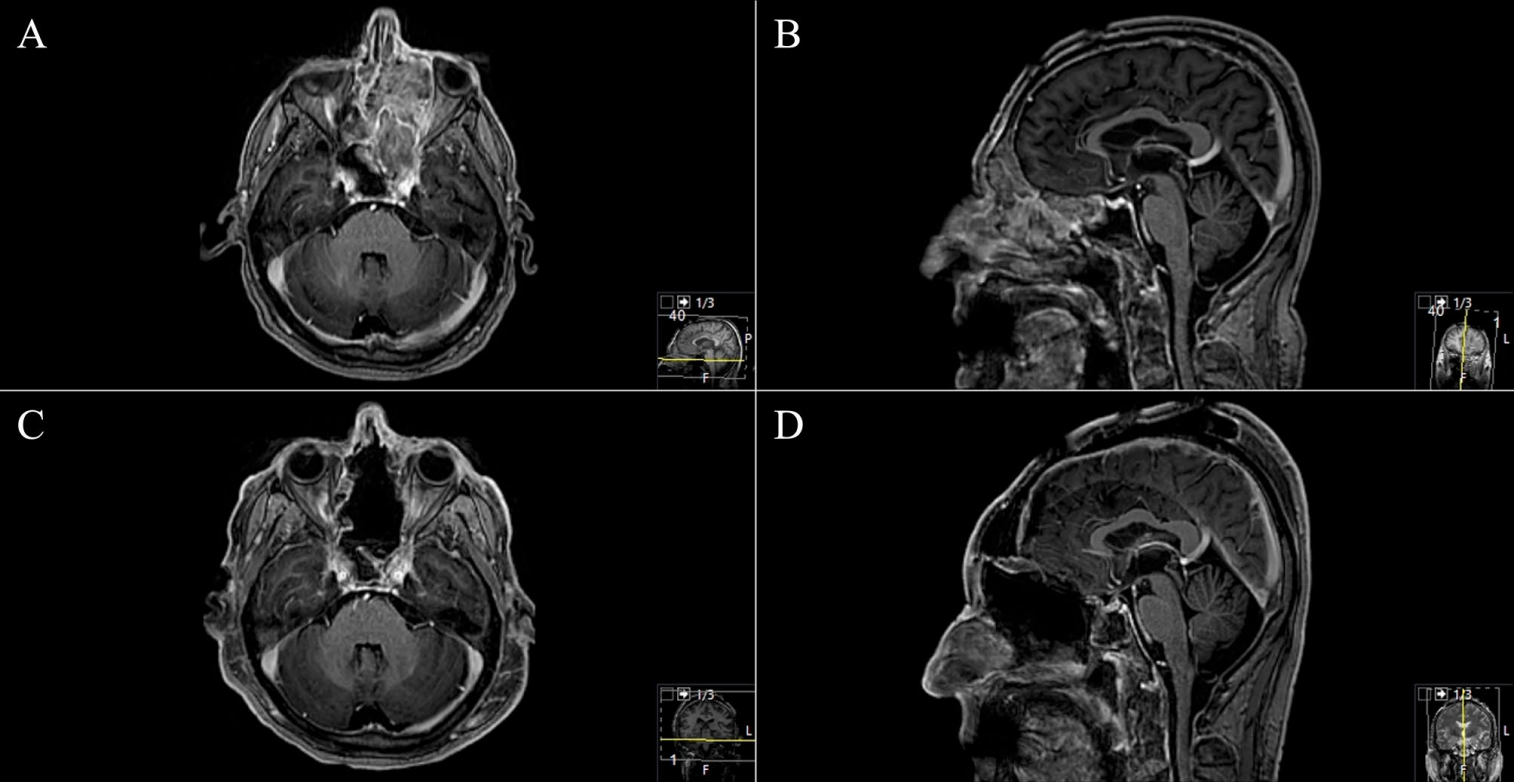
Exemplary case 2: a 70-year-old male patient presented with recurrent left eye inflammations and swelling over medial canthus. Ophthalmic examination revealed obstructed nasolacrimal ductus. (**A**,**B**) Preoperative T1-weighted gadolinium-enhanced MR imaging showing sinonasal and left orbit-infiltrating tumor. Ethmoidal and sphenoid sinus are completely infiltrating. Left bulbus is suppressed. (**C**,**D**) Postoperative MR control showing successful resection. Orbit, sphenoid and tuberculum sellae reconstructions were performed with split-bone graft, galea-periosteum flap and fibrin glue. Pathologic findings revealed metastasis of malign melanoma. The patient underwent endoscopic transnasal, sublabial reresection in the subsequent course.

**Figure 3:**
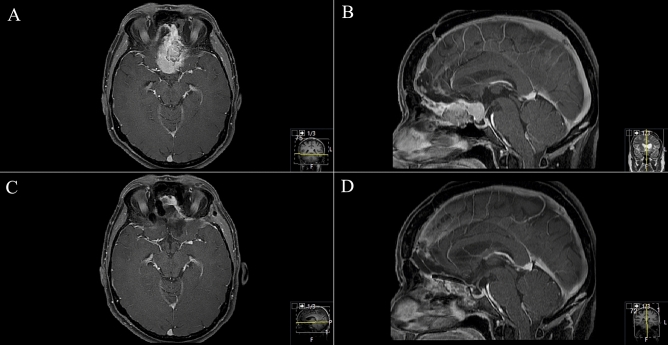
Exemplary case 3: a 60-year-old female patient with left amaurosis and right progressive visual deficit after two-timed resection of transbasal meningotheliomatous meningioma WHO grade I through a bifrontal craniotomy. (**A**,**B**) Preoperative T1-weighted gadolinium-enhanced MR imaging showing frontobasal meningioma with olfactory grove, ethmoidal and sphenoidal sinus and intrasellar affection. (**C**,**D**) Postoperative MR control showing Simpson II resection. Extradural clinoidectomy was performed in this case as well. Reconstruction was performed with split-bone graft and galea-periosteum flap. The patient recovered well from the procedure and underwent radiotherapy after multidisciplinary tumor board discussion.

**Figure 4:**
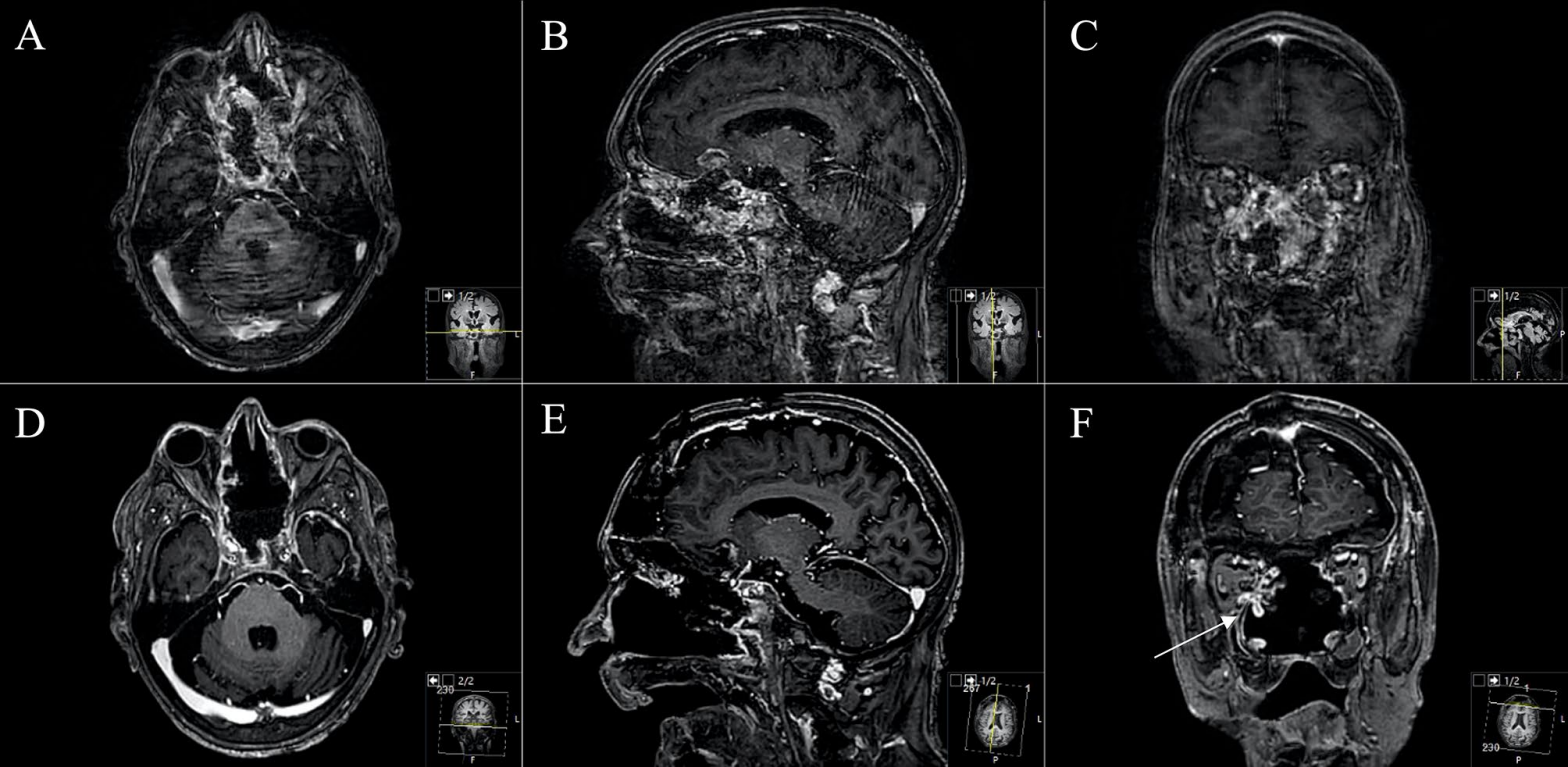
Exemplary case 4: a 78-year-old male patient with bilateral amaurosis, anosmia and psychomotoric disorders. Known sinusinal intestinal adenocarcinoma after endonasal biopsy. Radiotherapy was already carried out. Transnasal microscopic tumor debulking was performed a priori. (**A**–**C**) Preoperative T1-weighted gadolinium-enhanced MR imaging showing the huge infiltrating transbasal tumor with right orbital destruction and maxillary sinus affection. Reconstruction was performed with split-bone graft, galea-periosteum flap and collagen-based dura graft. No postoperative CSF leak occurred. (**D**–**F**) Postoperative MR control showing subtotal resection. F Small tumor remnant in the right periorbital.

**Figure 5:**
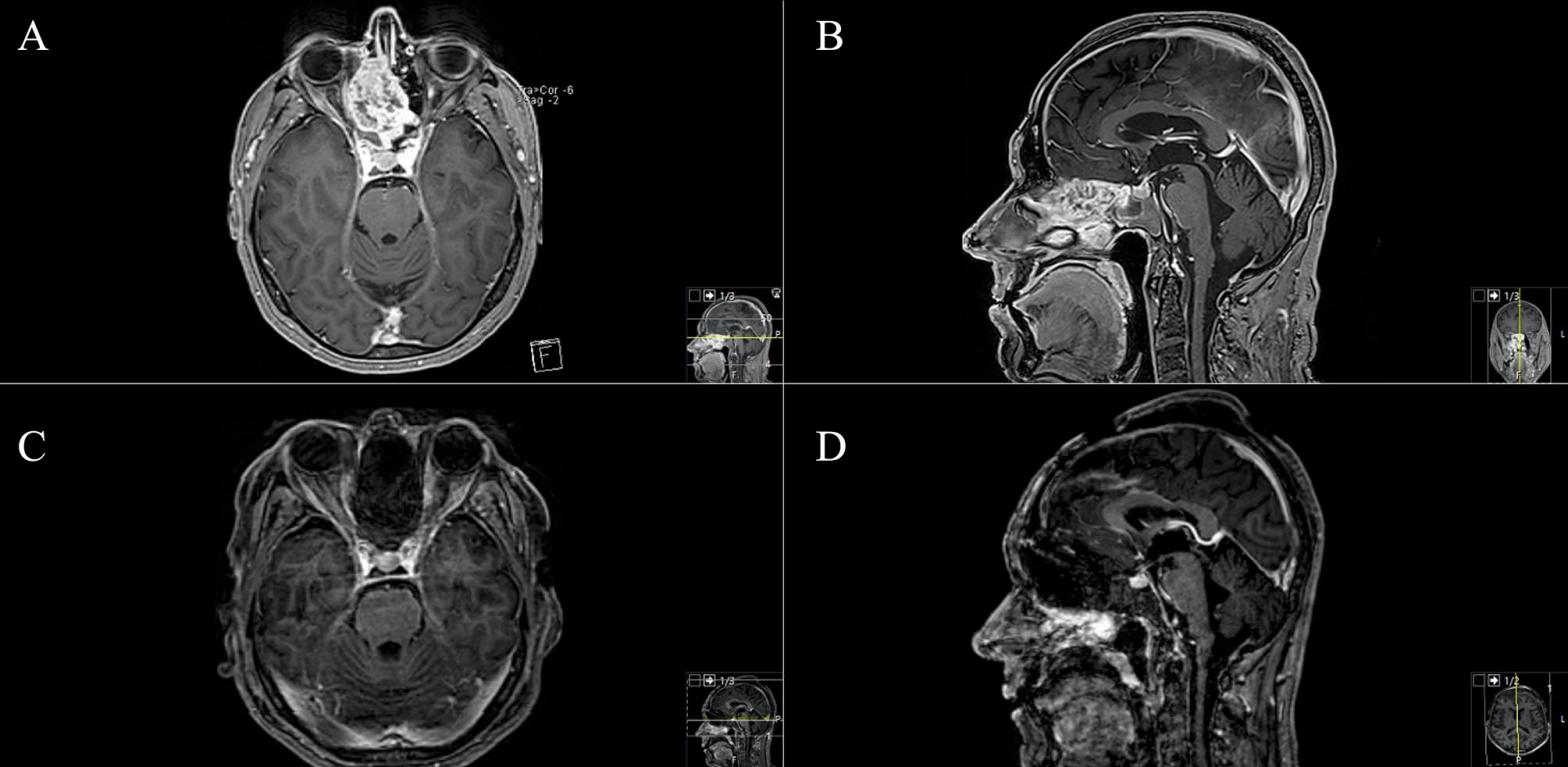
Exemplary case 5: a 64-year-old male patient with anosmia, rhinorrhoea, epiphora and headache. Transnasal biopsy revealed sinusinal intestinal adenocarcinoma. (**A**,**B**) Preoperative T1-weighted gadolinium-enhanced MR imaging endonasal tumor with transbasal infiltration into sphenoid sinus, cribriform plate and dislocation of right periorbit. (**C**,**D**) Postoperative MR control showing subtotal resection with questionable tumor remnant at inferior medial orbital wall. Tumor-infiltrated medial orbital walls and orbit roofs were resected. Reconstruction was performed with split-bone graft and galea-periosteum flap. Titan mesh was used as well. A second-step endoscopic procedure was performed for oncological gross total resection.

Histopathological analysis revealed four (22.2%) cases with WHO°I meningiomas, one (5,6%) metastasis, three (16.7%) adenoid cystic carcinomas, one (5.6%) invasive squamous cell carcinoma, one (5.6%) papillary transitional carcinoma, three (16.7%) sinonasal intestinal-type adenocarcinoma and five (27.8%) esthesioneuroblastomas). All TBAs were level 0 TBAs according to Feiz-Erfan et al.^5^ (Figs. 1, 2, 3, 4, 5). The most common tumor infiltration and destruction occurred into the orbit/orbital fissure/optic canal in 13 (72.2%) cases, followed by infiltration into the planum sphenoidale in ten (55.6%) and into the frontal sinus in nine (50.0%) cases (Table 2). The most commonly used technique for skull base reconstruction was creating a split bone graft and dural plastic via galea-periosteum-flap in 16 (88.9%) cases, respectively. Facia lata, Fibrin glue and collagen-based graft were used in two 11.1%, respectively (Fig. 4). Titan mesh was only used once.

**Table 2:** Skull base tumor infiltration and performed reconstruction techniques.

Skull base involvement/infiltration % (N)	Total (18)
Ethmoidal cell	44.4% (8)
Planum sphenoidale	55.6% (10)
Orbit/orbital fissure/optic canal	72.2% (13)
Crista galli	1.3% (6)
Endonasal	33.3% (1)
Frontal sinus	50.0% (9)
Sphenoidal sinus	27.8% (5)
Maxillary sinus	22.2% (4)
Pterygoid fossa	11.1% (2)
**Dural/bone reconstruction technique**	
Split graft	88.9% (16)
Galea-periosteum flap	88.9% (16)
Fascia lata	11.1% (2)
Fibrin glue	11.1% (2)
Titan mesh	5.6% (1)
Collagen-based graft	11.1% (2)

### Extent of resection, outcome and surgical complications

Gross total resection was achieved in 85.8% throughout all entities. Regarding meningiomas, Simpson grade I and II resection rate was 100.0% (Fig. 3). In order to achieve oncological total resection, a neck-dissection and a second-step transnasal endoscopic procedure (Fig. 2) were performed in three other cases. In one case an orbital exenteration was performed additionally.

Postoperative total complication rate was 33.3%. Venous congestion (11.1%) and one complication of pneumocephalus were classified as minor complications, as there was no necessity of urgent intervention. One complication of wound healing disorder and one of shunt-dependent hydrocephalus (5.6%, respectively) occurred in further follow-up. Therefore, rate of postoperative subsequent moderate-to-severe complications was 16.7% (Cerebrospinal fluid (CSF) leak 11.1%, chronic subdural hematoma (SDH) 5.6%).

Complication dependent re-intervention rate was 38.9% (Table 3). A revision due to CSF leak was necessary in 11.1% and due to haemorrhage in 5.6%. In one case, we performed a pedicled latissimus dorsi flap plasty^24^ (5.6%) due to wound healing disorders with abscess and consecutive bone ex- and implantation (5.6%).

**Table 3:** Extent of resection and postoperative outcome.

Postoperative presentation % (N) or mean/median (SD/range)	Total (18)
Complete resection rate (Simpson grade I + II and GTR)	85.8%
Extent of resection in case of other than meningioma	GTR 57.1% (8/14) STR 33.3% (6/14)
Simpson grade I–IV in case of meningioma	I 75%% (3/4) II 25% (1/4)
Intraoperative complications	Vascular (ACA injury) 5.6% (1)
Postoperative complications	*Minor complications* Pneumocephalus 5.6% (1) Venous congestion 11.1% (2) *Major complications* CSF leak 11.1% (2) SDH 5.6% (1) During follow up: WHD 5.6% (1) Hydrocephalus 5.6% (1)
Postoperative interventions In further course	Revision due to CSF leak 11.1% (2) Revision due to haemorrhage 5.6% (1) During follow up VP-shunt 5.6% (1) Bone explant/implant 5.6% (1) Pedicled latissimus dorsi flap plasty 11.1% (2)
New permanent neurological deficits	Psychomotoric disorders 11.1% (2) Dysphagia 5.6% (1)
Postoperative KPS	80.0% (20–100)
Follow-up time in months	9.8 (1.2–71.8)

**Figure 6:**
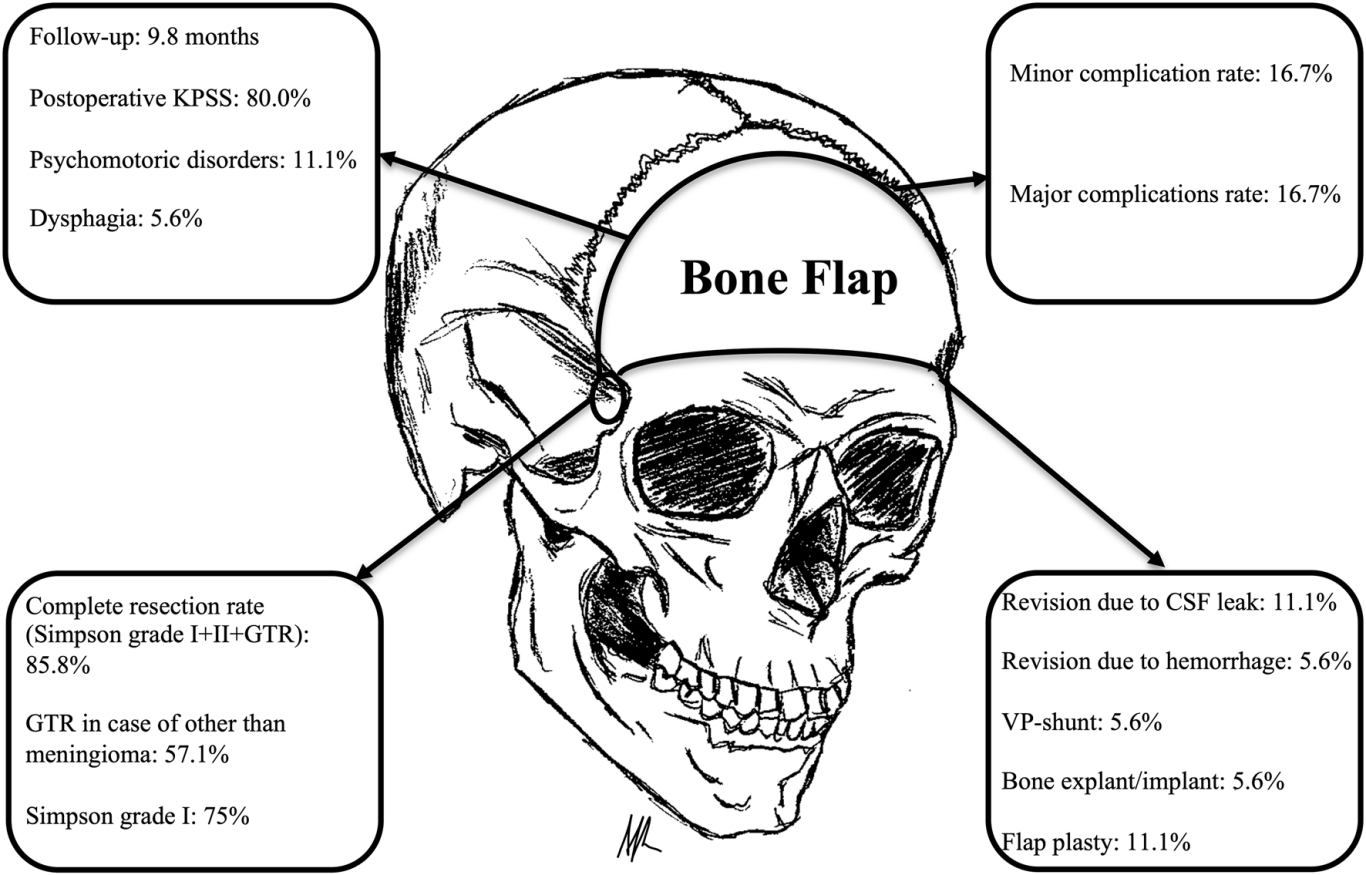
Illustration of classic basal bifrontal craniotomy for transbasal approach, complete resection rates, postoperative outcome and complications.

Median follow-up was 9.8 (1.2–71.8) months (Fig. 6).

## Discussion

**Figure 7:**
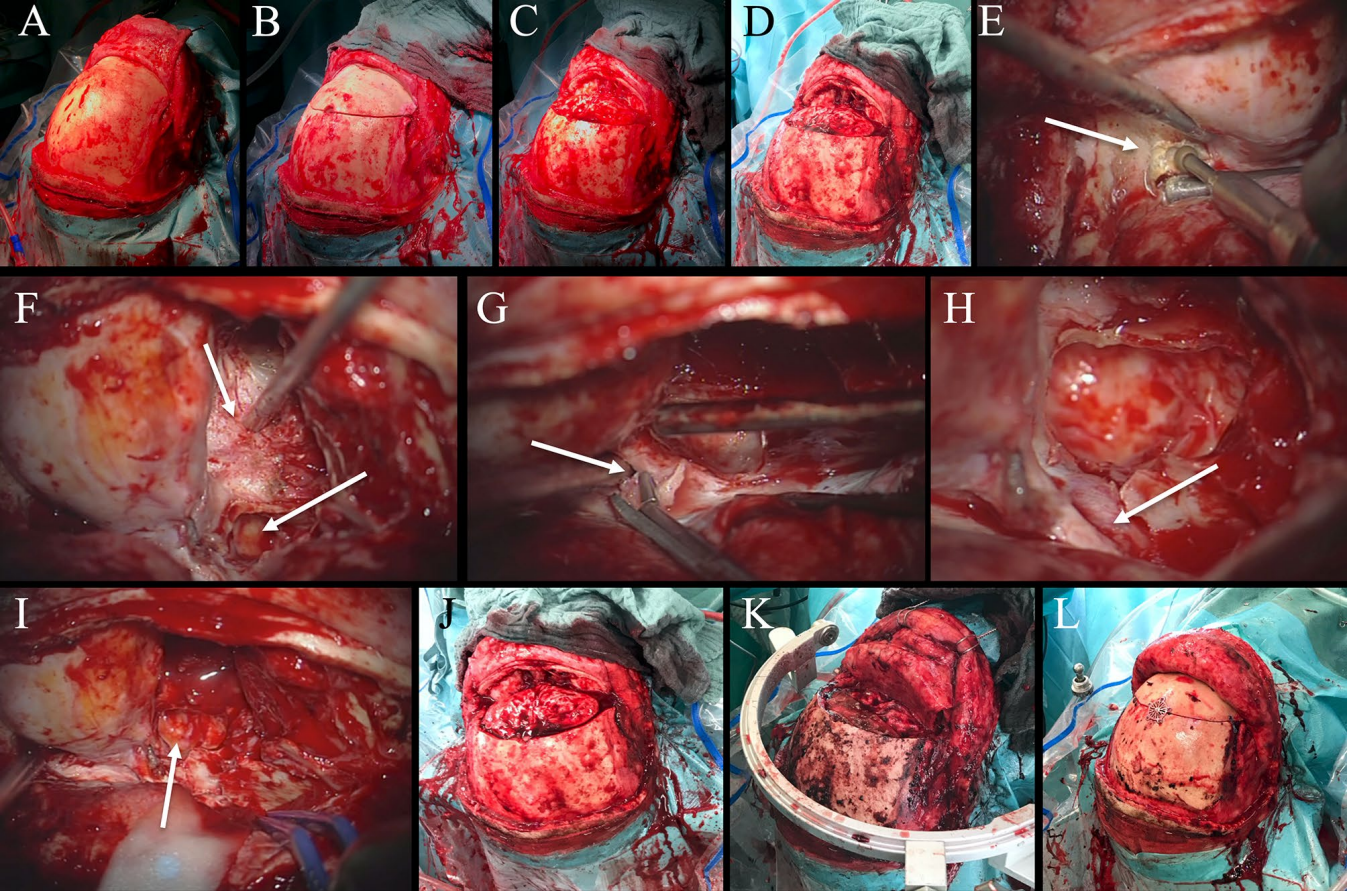
Illustrative case of classic transbasal approach. (**A**) Typical skin incision and preparation of a galea-periosteum flap for later reconstruction. (**B**) Bifrontal craniotomy. (**C**,**D**) Macroscopic view after bone flap elevation. (**E**) After initial tumor debulking further exploration by resection of lateral sphenoid wing. (**F**) Periorbit and left maxillary sinus exposed. (**G**) Removal of optic strut. (**H**) Exposure of opticocarotid triangle. (**I**) Subfrontal, transethmoidal, transsphenoidal and transmaxillary exposure. (**J**) Macroscopic overview after complete tumor resection. (**K**) Anterior skull base reconstruction is performed by galea-periosteum flap. (**L**) Bone flap re-implantation using thin flaps and closure.

In our patients, TBA is a technically demanding but satisfying operative technique to expose a deep, transbasal-lying lesion in the anterior skull base, the midline structures and the interhemispheric fissure. In the majority of cases, complete resection without a new neurological deficit could be achieved. Complete resection rate (Simpson grade I + II and GTR) was 85.8% with a total complication rate of 33.3%. Rate of postoperative complications requiring intervention was 16.7%. No surgery-related death occurred. According to the proposed classification by Feiz-Erfan et al., all performed TBAs were level 0 basic TBAs (Fig. 7).

The TBA is a widely used anterior, midline and craniofacial approach with a variety of modifications and extensions. Derome initially popularized TBA for midline pathologies of the anterior skull base^6^. One of the big advantages is the ability to avoid a transfacial procedure and to allow craniofacial resection by a craniotomy-only technique^11^.

Many attempts for a classification have been made^13^, for example, to divide the approach into 3 subgroups: “traditional,” “extended” and “extensive”^11^. Traditional TBA has been defined as a simple bifrontal craniotomy. The “extended” TBA includes the removal of medial supraorbital rims between the supraorbital notches, whereas the “extensive” TBA includes complete removal of the supra-orbital bar. However, surgical techniques have developed further, and other extensions or modifications of already existing variations have been added to the conglomeration of TBAs. Feiz-Erfan et al. summarized these modifications and provided a new classification system to unify the TBA terminology of the past decades^5^. They proposed four levels, ascending in extension: level 0 TBAs, as consistently performed by us, consists of a classic bifrontal craniotomy without facial osteotomies. The other classes encompass more aggressive osteotomies up to canthal ligament resection with canthopexy, such as in level 3 TBAs.

Anyway, many authors have described their techniques and style of approach, where complication rates have ranged from 30 to 50%, but they have also described rates over 70%^5,11,18,25–32^. Our complication rate of 33.3% is in the lower bound of those findings. One should notice that we resected all lesions by level 0 TBAs (Figs. 1, 2, 3, 4, 5). Other authors have described complete resection rates between 80 and 93%^16,25,29^, which are congruent to our rate of 85.8% using a relatively simple technique. We did not see any urgent need for extensive osteotomies. On the contrary, we preferred second-step transnasal endoscopic re-resections in further clinical courses to achieve oncological R0-resection in 16.7% (3) in order to avoid cosmetic deformity and higher perioperative morbidity. With targeting the lesions with another angle of view by more minimal-invasive approached such as above-mentioned endonasal techniques in a second step, the TBA may be kept as simple as possible. In general, implementation of minimally invasive approaches wants to benefit from endoscopy^33–35^, such as the endoscopic endonasal approach. However, literature shows that only certain cases are suitable and extent of resection is only 66.7% for an endonasal endoscopic approach in case of midline meningiomas of the anterior skull base^34,36^. Open transcranial approaches for olfactory groove meningiomas, for example, still result in higher rates of total resection with lower postoperative CSF leak rates^33^. Purely endoscopic or endoscopy assisted resections via transnasal or transcranial routes have been popularized for some time offering minimal invasive visualization of the skull base region^34,37^. This trend has not peaked into a paradigm shift as extent of resection and CSF leakage rates have been described to be unfavorable compared to classic skull base approaches^33–35,37^, which is why a TBA should be performed primarily as done in our series.

Regarding complications, CSF leaks are considered the most common ones through TBAs, especially after dural reconstruction^10,38^. Postoperative leaks may be controlled with lumbar drainage as we did regularly. Otherwise, revision is necessary. In our series, a revision due to CSF leak occurred twice (11.1%), which is comparable to the findings of, for example, Obeid et al. with 20% or Raveh et al. and Kurtsoy et al. with 4.8% and 6.0%, respectively^29,32,39^. Regarding infections, incidence is actually low^10,16,18,21,32,38^. They may occur in the case of larger tumours and longer operative times requiring surgical revision with bone flap explantation and plastic flap reconstruction, as with one of our patients.

Regarding postoperative anosmia, the sacrifice of olfactory nerves is often necessary during a TBA. Researchers have attempted to minimize this loss of function. Spetzler et al. described a cribriform plate osteotomy to preserve olfaction^28,40,41^. In selected cases, preservation of more than 90% was reported, which other authors confirmed as well^5^. However, this may be practicable if transbasal infiltration just occurred to a certain extent. In the case of huge infiltrating tumours, such as nasosinal adenocarcinomas, esthesioneuroblastomas or cribriform and endonasal-infiltrating olfactory groove meningiomas, preservation of olfaction remains hardly possible. Some groups reported experimental methods. Matano et al. described a “fibrin-gelatine fixation” method to preserve olfactory function by dissection of the olfactory bulbs from the frontal lobe and by fixing them to the frontal base surrounding bulbs using a thin slice of gelatine sponge with fibrin glue. Anosmia occurred in 4 patients (8.8%), which was transient in two (4.4%) and permanent in the remaining two (4.4%) of a series of 45 olfactory groove meningiomas^42^.

More aggressive approaches to anterior cranial base lesions are on the rise and advocated by some. Although transfacial and transmaxillary approaches may provide reasonable surgical access, they may also be associated with a higher incidence of cosmetic deformity, CSF leak, infection and mortality^10,19,43–45^.

However, despite the above-mentioned heterogenous surgical philosophies, attention should be paid to the fact that we performed relative standardized TBAs without extra osteotomies or modifications in order to keep surgical processes simple and comparable. Of course, the size of craniotomy and the exact position depended on the location and size of the lesion; nevertheless, our results showed satisfactory postoperative findings regarding EOR, perioperative comorbidity and postoperative outcome. Therefore, we recommend performing TBAs if indication is comprehensible. TBAs still remain technically demanding, but if surgical considerations are kept simple and straightforward, this approach turns out to be a cosmetic and functionally justifiable technique for different transbasal tumours.

### Study limitations

As it is a retrospective case series, it is not possible to draw causalities with respect to clinical outcome. Nevertheless, we implemented detailed clinical examination, including scores on functional performance, and a standardized follow-up protocol based on a certified neurooncological board into our clinical workflow. In our study, we did not focus on the long-term outcome of different tumor entities but on the surgical approach and perioperative outcome.

Besides its retrospective nature, the analysed patient collective suffers from histological heterogeneity. The presented histological entities are quite mixed. They include benign and malignant lesions. As we decided to to determine the outcomes of the technique and focus on the TBA itself we have accepted pathological heterogeneity to discuss this problem from a more surgical and technical point of view.

## Conclusions

This study shows that modifications and osteotomy extensions of the classic TBA are unneeded. Complete resection can be performed under functional and cosmetic-preserving aspects. Second-step procedures such as transnasal approaches may be taken into consideration to avoid higher perioperative morbidity of more aggressive TBAs, if necessary. Surgical considerations should be kept simple and straightforward.

## Data Availability

The data for this manuscript can be obtained from the author upon reasonable request.
